# Experiences of families when a member is in forensic psychiatric care: a phenomenologically based thematic interview study

**DOI:** 10.3389/fpsyt.2025.1564591

**Published:** 2025-04-22

**Authors:** Gustav Björnberg, Ulrica Hörberg

**Affiliations:** ^1^ Department of Health and Caring Sciences, Linnaeus University, Växjö, Sweden; ^2^ Research Department, Region Kronoberg, Regional Forensic Psychiatric Clinic in Växjö, Växjö, Sweden

**Keywords:** experience, family perspective, forensic psychiatry, phenomenology, qualitative methodology

## Abstract

**Introduction:**

This article focuses on the meaning of being a family when a member of the family is cared for in forensic psychiatric care. The latter operates at the crossroads of psychiatric services and the legal system, making it a highly complex area of care. Families play a vital role in supporting recovery from mental illness, however, when a family member has committed a crime and suffers from severe mental illness, pressure and stress of a complex nature are felt by the family. The aim of this study was to describe the lived experiences of families when a member is in forensic psychiatric care.

**Material and methods:**

Seven family interviews (15 participants) were conducted and analyzed with a Reflective lifeworld approach in this phenomenological study.

**Results:**

The findings reveal four themes; Being constantly affected by uncertainty; A struggle to maintain family relations – overcoming barriers in the form of restrictions; Trying to help despite inherent powerlessness and It is not just the patient who needs to heal when the family is wounded.

**Conclusion:**

A family with a family member who is being cared for in forensic psychiatric care have support needs that are not fulfilled, even though these have been highlighted from several different perspectives. Healthcare professionals might be able to provide substantial support to the family by using simple yet effective methods, such as active listening and empathetic communication. By fostering an open dialogue and understanding, caregivers can help bridge gaps and facilitate better outcomes. A family-oriented practice should thus become a fundamental component of caregiving strategies in forensic psychiatric care.

## 1 Introduction

This article focuses on the meaning of being a family when a member of the family is cared for in forensic psychiatric care. The latter operates at the crossroads of psychiatric services and the legal system, making it a highly complex area of care (1). The legal regulations that control the provision of forensic psychiatric care differ across the world (2, 3). Forensic psychiatric care in Sweden is based on the Health and Medical Services Act (4) while also being influenced by compulsory care legislation (5, 6). Approximately 3,300 patients were cared for in both open and closed forensic psychiatric care settings with different security classifications in Sweden in 2022 (7). Forensic psychiatric care is complex and multifaceted, combining the aim to protect society from the danger presented by the patient with the goal to provide care that leads toward the patient’s recovery and freedom (1, 8) Forensic psychiatric care is provided in each county in Sweden, where the security classification of the clinics can differ. It is not uncommon for patients to be referred to regional clinics with higher security levels, where they can spend several years before returning to continue their care in their home county (9).

Families play a crucial role in the recovery of individuals with severe mental illnesses, providing significant moral support and serving as a motivating force throughout the rehabilitation process (10, 11). However, when a family member has committed a crime and suffers from a severe mental illness, the family dynamics often become complex, leading to considerable emotional, financial, and psychological burdens (12, 13).

Although both patients and healthcare professionals acknowledge the importance of maintaining family relationships and utilizing family support in forensic psychiatric care, this practice remains relatively uncommon in forensic psychiatric care in Sweden (14, 15). The National Board of Health and Welfare emphasizes the importance of involving families to a greater extent in forensic psychiatric care. However, surveys conducted by this authority show that families and relatives often feel excluded from the care process (16, 17). This was exemplified in Rowaert et al. (18) who identified that family members face barriers in several areas when trying to support one another, including involvement in care, information sharing, visiting procedures, transitions between wards, and communication with psychiatric and judicial professionals. Additionally, families have highlighted the need for social support for themselves during the forensic psychiatric care process. Despite these needs, adequate interventions for families are often lacking in forensic psychiatric services (19, 20). Addressing these challenges, as shown by Pearson and Tsang (21), could significantly improve the situation for families without substantially increasing costs.

There is a need for a family-oriented practice in forensic psychiatric care, and in order for this to be possible there must be an interest in understanding the experience of being a family when a member of the family is undergoing forensic psychiatric care (15, 22). The research focusing on the phenomenon” being a family when a member is cared for in forensic psychiatric care” is insufficient, and knowledge of qualitative perspectives within a Swedish context is virtually non-existent. It is thus important to gain a greater understanding of being a family under such circumstances, and to identify their support and help needs, as well as to gain knowledge of how patients and family members perceive their internal and external relationships and interactions with the forensic psychiatric care services.

## 2 Aim

The aim of this study was to describe the lived experiences of families when a member is in forensic psychiatric care.

## 3 Materials and methods

This study is a qualitative interview study grounded in phenomenological ontology and epistemology, which provide opportunities for a greater understanding of a phenomenon. The aim of this study is to describe and gain a greater understanding of the phenomenon: being a family when a member is in forensic psychiatric care. The choice of method has been made based on the ambition to explore and describe meanings of the phenomenon based on the informants’ lived experiences and perspectives.

A thematic meaning-oriented analysis approach according to Lindberg et al. (23) has been used. This approach is grounded in the ontology and epistemology of Reflective Lifeworld Research (RLR) by Dahlberg et al. (24). RLR draws from the rich philosophical underpinnings of phenomenology, including Husserl’s lifeworld theory (25) and the theory of intentionality (26) as well as Merleau-Ponty’s theory of the lived body (27) and the ontology of “the flesh of the world” (28). The methodological principles of openness, flexibility and bridling have been adopted during the research process, and the concepts of objectivity, validity, and generalizability have been used to assess the quality of the study.

### 3.1 Data collection and participants

A total of 15 informants from seven families were interviewed based on the patients’ definition of their families. There were different family constellations from four different forensic psychiatric clinics in Sweden. All of the patients were male. Two female researchers in the research group who had rigorous experience of conducting qualitative interviews in the context of psychiatric care conducted the interviews. The participants had no established relationship with the researches prior the commencement of the study. The interviews were conducted with one patient and at least one family member present simultaneously, aligning with the phenomenological perspective of viewing the phenomena as a cohesive “family”, rather than a collection of fragmented parts of “family members”. Open and circular questions were used in the interviews with the support of an interview guide, which aimed to clarify the relational interplay within the family and promote reflection on differences and similarities in the family members’ descriptions. The same opening question which was used in all the interviews was: What it is like being a family when one of you is being cared for in forensic psychiatric care? The interviews were based entirely on the voluntariness of the informants, who could withdraw from the interview at any time without having to explain the reason why. The interviews, which lasted between 45-60 minutes, were audio-recorded and then transcribed. The interviews were conducted in both inpatient and outpatient forensic psychiatric care across all security levels. Calm rooms were used as a setting for all of the interviews. The data material has after transcription been stored in a safe without access by anyone not involved in the project.

A purposive selection procedure with the aim of obtaining variations in family characteristics and thus more variable data was applied after the researcher group received notification of interest from potential informants. The inclusion criteria for the patients of the families are described below.

Individuals eligible for the study included those who had been receiving care for at least three months in accordance with the Act on Compulsory Psychiatric Care (5) and/or the Act on Forensic Psychiatric Care (6) including both inpatient and outpatient forensic psychiatric care. The specialist physicians responsible for the patients’ medical care first evaluated whether a patient’s health condition allowed them to participate in an interview. If deemed appropriate, the patient was asked if he would like to participate, and the patient contacted their family if they would like to participate in the family interview. The family members provided explicit consent after receiving the same information about the voluntary nature of the study and the option to withdraw at any time. All the participants were informed of the goal of the research project before giving consent.

The family constellations participating in the interviews are stated in **Table 1**.

**Table 1:** Patient and Family member roles.

Patient role (all male)	Family member role
Son	Father
Brother	Sister
Brother	Brother
Friend	Friend/Support person^1^ (male)
Son	Mother and Stepfather
Grandchild	Grandmother
Uncle	Niece

The participants varied from 19-65 years of age.

### 3.2 Data analysis

The interviews were analyzed using a meaning-oriented thematic analysis (23) grounded in a reflective lifeworld research approach (29). The analysis aimed to describe themes of the meaning of the phenomenon “being a family when member is in forensic psychiatric care”. The analysis contributed to a higher level of abstraction based on the data obtained from the interviews and resulted in themes of meanings supported by quotes from the interviews. An open and scientific strategy was applied in the analysis process, which in a phenomenological reflective lifeworld approach entailed having a reflective attitude with an openness to discover and understand something new. A reflective attitude also entailed slowing down the understanding process and attempting to not understand too quickly or unreflectively (29).

The analysis was conducted using a meaning-seeking approach (23). Firstly, the interviews were read through several times in order to gain a broad perspective of the material at hand. Meaning units in the text were then identified and their meanings were described (meaning unit is a part of the text that consists of meanings of the phenomenon).

Similarities and differences served as the basis for organizing related meanings into clusters, which were subsequently grouped together. These clusters and their patterns of meanings were then further related to each other and emerged into four overarching themes of meanings. A flexible approach was employed during this process whereby themes, patterns, and meaning units were continuously tested against each other and the phenomenon. Four themes emerged during this process, and together those themes describe the phenomenon.

### 3.3 Ethical considerations

Research ethical considerations were carried out prior to the start of the research project, the study was approved by the Regional Ethical Review Board at Linkoping University, Sweden (reg. number 2011/228–31). The interviews were judged to potentially pose a risk of arousing difficult feelings. The research team who conducted the interviews has expertise in psychiatric care with extensive experience in caring conversations as well as conducting interviews with individuals and families who are in situations marked by mental illness or vulnerability. There was an awareness that the patients’ mental state could make it difficult to participate in the family interviews, whereupon sensitivity and responsiveness was emphasized.

## 4 Results

The analysis resulted in four themes of meanings that together describe the phenomenon “Being a family when a member is in forensic psychiatric care”. The families’ experiences are presented in themes as follows:

1. Being constantly affected by uncertainty.
2. A struggle to maintain family relations – overcoming barriers in the form of restrictions.
3. Trying to help despite inherent powerlessness.
4. It is not just the patient who needs to heal when the family is wounded.

### 4.1 Being constantly affected by uncertainty

Being a family when a member of the family is in forensic psychiatric care is experienced as unclear and fragmented. A lack of context and experiencing emotions of uncertainty dominate the present, which makes it difficult to form a meaningful future as a family. The barriers to freedom can seem impossible to conquer for a fractured unit (family), engendering feelings of being hindered from the possibility of attaining desired autonomy.

Planning for the future as a family is extremely challenging due to the lack of a clear time frame and the unpredictable nature of the care processes. Forensic psychiatric care does not provide the same predictability as is found in prisons where the length of the sentence is pre-determined, which makes it possible to plan for life after release. It instead appears as unclear and fragmented where decisions, such as when a patient is transferred to another ward, psychiatric setting or region, create physical distance and prolong detention. Moreover, these decisions, which the caregiver has the responsibility to take, are taken without consideration for the family’s situation.

*“Well, they can come tomorrow and say that I’m going back to X-city, for example, they can do that. That the doctor has decided that, and it’s not so damn funny. And then I know how it’ll be, I’ll be there for a while and then come back here and then it’s back to square one again”* (Patient)

The lack of sufficient information causes a constant state of worry and doubt, increasing the uncertainty and impeding mutual support. Healthcare staff may withhold information, further exacerbating distrust toward the forensic psychiatric care system. The restrictions that the care applies become a symbol of what stands in the way of an existence in harmony. A father tries to put words into what it is like not to receive information about his own son.

*“And then I called and talked to them (healthcare professionals) and asked how he was because I’d heard he was like that when he got back. Anyway, they couldn’t say anything, they couldn’t get consent. So, there was no information at all. It’s like getting a slap in the face. Yes, I heard he was psychotic and in a bad shape and I didn’t really know how to deal with that. And you (the patient) could call me five times a day without remembering.”* (Father)

Feelings of worry emerge within the family when contemplating the future. Thoughts of returning to a previous life and social circles can generate negative memories. Prevailing rules that the patient must move back to the county where the patient used to live when the regional psychiatric inpatient care is over can create a fear of encountering previous criminal acquaintances and relapsing into negative patterns in a way that affects the whole family. A patient talks about his fears when facing a future life in freedom.

*“After all, you wonder when you get out, when you are discharged from care, what it will be like then, how people will react over time, it’s that kind of thing … you’re very worried about. You read in newspapers about people who have encountered trouble after serving their sentences, so you’re afraid of that…”* (Patient)

### 4.2 A struggle to maintain family relations – overcoming barriers in the form of restrictions

The relationships between the family members are hindered from being naturally occurring. The loyalty, duty and responsibility for each other is put to the test when trying to overcome the barriers that stand in the way of maintaining relationships within the family.

Family relationships become more superficial for a number of reasons. These include: profound conversations not occurring anymore due to a family member’s mental illness, while large geographical distances and prevailing security restrictions force the interactions within the family to take place in supervised and occasionally more clinical environments.

*“We lose a lot, we’re not able to talk intimately at all.//… We’re going to town later and you (the patient) must have two staff members with you, it won’t be the same. To sit here instead in a visiting room at an institution, you don’t really want to do that either…”* (Father)

More profound conversations and intimate companionship can instead take place during short-term releases or in settings with less restrictive security classifications. These moments serve as valuable opportunities for families to reconnect. Spending time together at home or during a stroll in nature is experienced as a more natural and meaningful way for family members to strengthen their bonds and relationships. Lacking this opportunity is associated with powerlessness, disappointment and failure. A patient and his brother discuss what this might look like in reality.

*The brother: Now we get to talk and maybe meet for just one day. And that’s not really okay, I don’t think. They (the staff) don’t help for five cents with family and such, I don’t think so. I do not think so!*

Patient: It was the same thing when they (his family) went all the way down from “*my hometown*” and then they weren’t allowed to enter. So, it’s just sickening.

*Interviewer: To visit you?*

*Patient: Yes.*

*The brother: Yes, we had a scheduled appointment, but then they were short of staff. And they can’t really blame that. We’d, as it were, driven more than 120 miles (200 kilometers).*

The possibility of talking on the telephone bridges the physical distance and gives the opportunity to approach each other in a way that goes beyond the concrete restrictions. Calling each other without a specific reason appears to be an action that creates presence and maintains the family as a living unit. The mobile phone and the landline phone^2^ are not only communication tools but also symbolize different aspects of companionship. The mobile phone, appears as a privilege, providing not only technological convenience but also opening doors to alternative forms of contact via social media. These mediums broaden the opportunities for the family to maintain a continuous interaction that extends throughout the day. Two parents talk about what it can be like when being deprived of this opportunity.

*The mother: Yes, we were very limited and at the same time it affected us a lot, because our son had access to the internet and we were there, messaging via Facebook and Messenger and that.*

*The father: It’s easier to talk to people.*

*The mother: But I thought it was difficult in that particular period. It was hard for our son because he had nothing to do here and he didn’t know what to do.//. trying to find something to replace this here, instead of sitting and talking to his friends via the internet which didn’t work, so he had to find something else to occupy himself with…//… so that we could talk with each other yes … send greetings to him from those we have met, those who greet him via us and via him to them…*

The restrictions that the care implies appear as a form of power, which can deprive the family of its true nature and relationships with each other. The duty, loyalty and responsibility for each other within the family is put to the test requiring continual efforts to maintain relationships, where every handling and decision affects the outcome.

*“It was probably more that I persisted and called and called and called, and sometimes you called, but mostly it was me calling. But I agree with my brother, it was indeed … yes, the exact word, fumbling. Because I had no idea, my brother had no idea, we just … end up in a big black hole”* (Sister)

### 4.3 Trying to help despite inherent powerlessness

The suffering becomes tangible in the family when the burden of the long period of detention and the patient’s illness is shared, and they are unable to influence the situation. It is in the nature of a family to help each other, but the help is felt to lack importance in achieving the care goals, the family relationship is instead characterized by its ability to reduce the suffering that deprivation of liberty and mental illness create.

Helping each other is crucial when navigating through the uncertain and unpredictable existence. However, the helping is overshadowed by a prominent helplessness within the family. It appears difficult to understand and be aware of how someone feels and fares when their access to each other is restricted. It is also impossible to influence the most important issue, freedom and autonomy. The innate vitality in “helping” each other within the family is shown, for example, by supporting through conversations or by giving each other advice. Mutual help given within the family is often seen as more significant than the guidance and advice given by staff, and this is in spite of this type of help having an inherent powerlessness. In the following quote, a grandmother talks about how she helps her grandson express his opinions with staff when they are planning his care.

*The patient: No, so I’ve become, I’ve been a bit low lately.*

*The grandmother: Well, and then as I’ve felt that he was low and depressed ‘I have no rights to say anything’ and therefore I have joined in and said what I think he wanted to say. I’ve said it so many times to him (the patient) at meetings and such - to express yourself. That’s what it’s all about.*

Despite a shared suffering within the family, they can rejoice in each other’s success. The joy can take the form of a family member getting a job, overcoming drug addiction or getting a long-awaited leave.

Feeling safe is described as being necessary to be able to receive the help that care aims to provide. However, forensic psychiatric care often results in feelings of uncertainty in the family, while at the same time, the staffs’ care, honesty and interpersonal behavior can prove to be able to mitigate this uncertainty. For example, the feeling of being listened to can be strengthened when the staff listen to their opinions and wishes thus showing that there is a mutual endeavor to achieve something better.

*“Mmm, I thought it was great the support that we received from the staff who work here, especially those who took care of our son, to whom he turned so much, as he revealed himself to. It was great, they came to visit us at home too, with the staff here we could talk a little more about our son’s difficulties, that it’s sometimes hard for him and sometimes he thinks it’s hard to explain certain things. But then you can see these little signs when it’s like that, and I thought that was great”* (The patient’s mother)

### 4.4 It is not just the patient who needs to heal when the family is wounded

The family’s current existence is shaped by mishaps and traumatizing events, by mental illness, deprivation of freedom, and criminality, and also by its historical context. The family as a system has wounds that have not healed. To enable healing, family members may be excluded or replaced. Various events emerge in this dialogue that have created a wounded family system.

*Niece: Grandma was really sad and felt really bad.*

*Patient: I felt bad when she passed away, the last years I was out there at her home. It was awful, damn cancer. My mother had something in her throat, I know, because when she was about to eat, she threw it up again. I left because I had to catch the bus at a minute past six. Then she died at night. So, I cried like hell.*

*Interviewer: Then she was dead?*

*Niece: Yes, but grandma threw him out before, when he took drugs and had bad company, he couldn’t stay there anymore - and then she got sick and then she passed away. And then you (the patient) got an apartment that you set on fire.*

Living with “unhealed wounds” in the family system is experienced as making life more difficult. It is described as generating poor opportunities for a future where the family as a whole is once again part of society. The unhealed wound presents itself as a burden that does not go away by itself, but instead becomes deeply rooted in the family’s existence. For example, through a criminal lifestyle that the brothers below share with each other.

*Brother: You don’t have that, my mother said we should steal our own clothes. That’s how it’s worked, then it all goes seriously wrong. I think it’s awful.*

*The patient*: The whole family have been terrible criminals.

*Interviewer: How do you feel about it today when you think about it?*

*Brother: That’s why I have no contact with her. She doesn’t deserve my contact, not a chance.*

This burden of having unhealed wounds such as a criminal lifestyle in the family or fragile connections between each other can also cause difficulties for the family when participating in the care, for example, being involved in the care processes, cooperating with staff and contributing to positive consequences for caregiving and the medical treatment as a whole. This leads to feelings of being stigmatized as a family, who become a burden that is not able to contribute anything positive to the care or to the patient’s treatment. A father highlights in the example below how staff expected them to have a poor relationship with their son.

*Father: We didn’t really have a conflict, but that’s what the doctors thought we had, a conflict.*

*Interviewer: Between you?*

*Father: Between us, like.*

*Patient: We never had that.*

*Father: Nah, so we would sit and talk about what happened and stuff. So, he (the nursing staff) thought it would be like a war in that room, but it wasn’t, so he was shocked by it, but instead we talked about what had happened and why it happened.*

That staff are able to set aside time for the family is crucial for how the care is experienced by the family. This is not only because it inspires hope and aims to achieve improvements, but also because it can compensate for the conditions and opportunities and facilitate coping with the obscure and uncertain reality that the care entails. Doing this in a way that is genuine, i.e. by staff being there even though they do not need to, can show that the help and care is something that is done with good intentions. The following is a discussion that describes how honesty, humanity and genuine reciprocity contribute to a help that is appreciated by a patient and his support person (family member).

*Support person: Wonderful gentlemen.*

*Patient: Yes, of the old sort.*

*Support person: Yes, and they have called once a week both to me and to you. Checking how it’s going and stuff, so there’s been some support. That’s really good.*

*Patient: Yes, that’s what I think. I call them every day, but I didn’t need to now, they said. But otherwise now I have called them every day and talked a little. They have agreed that everything is fine, so it’s worked well.*

## 5 Discussion

The findings of this study shed light on the complex challenges faced by families navigating life when a member of the family is cared for in forensic psychiatric care. The central theme revolves around the persistent struggle with uncertainty and fragmentation, which affects every aspect of familial experience in this context. Moreover, the struggle to maintain family relationships amid the constraints of forensic psychiatric care emerges as a prominent and inherent powerlessness experienced by families in trying to navigate through the care system while trying to provide help for each other.

The prevalent theme of uncertainty and fragmented experiences of the care is similarly reported in earlier studies and papers, which mainly describe a lack of continuity in the care, a lack of information, distrust in the quality of care, and trouble in contemplating the present and the future (18, 30, 31). However, the present study adopts a unique approach by collectively interviewing families, thus departing from the common practice of conducting separate interviews with patients and their families. This approach reveals that the uncertain and fragmented nature of care extends beyond the immediate context of the care system, significantly affecting the entire family in all of its existence, both in the present and the future. The feeling of being underinformed is crucial for this experience and families find it challenging to convey information to each other in the family and also difficult to get information about the content of the forensic psychiatric care that is provided. This lack of information is closely linked to feelings of worry, doubt, and uncertainty, aligning with the findings in previous research (32, 33). Organizational and legal constraints within forensic psychiatric care appear to inhibit the dissemination of information. Families are heavily relying on the willingness of individual staff members to dedicate time to explain procedures and address their questions in this study, as well as in the study by MacInnes and Watson (34). The findings also shed light on how prevailing confidentiality regulations may impede the family members’ understanding of each other and the situation at hand. This finding resonates with the study by Sampson et al. (35), suggesting that such regulations can contribute to misunderstandings and erode trust in healthcare professionals. The complexity of confidentiality rules may obscure answers to the families’ inquiries, rendering them unclear and challenging to navigate. Greater knowledge about and comprehension of confidentiality among healthcare professionals seems to be of importance.

This study highlights that the maintenance of family relationships is challenging, because of several different factors. The foremost among these is the stringent security regulations^3^ in forensic psychiatric care (36), which hinder natural familial interactions. Additionally, the considerable physical distance^4^ inherent in Swedish forensic psychiatric care exacerbates the issue. Lengthy distances obstruct in-person meetings and hinder the intimate conversations vital for sustaining familial bonds. The relocation of patients to hospitals far from their families further exacerbates the prevalence of long-distance family relationships. Our study, in line with previous research, highlights the importance of and the challenges that forensic psychiatric care face in facilitating family interactions toward a more natural habitat (15, 22). Moreover, psychiatric illnesses significantly hamper communication, connectivity, and mutual understanding within families, compounded by associated burdens. Despite the evident need for support, our findings, together with those in previous studies, suggest a lack of adequate assistance from the care system in addressing these familial challenges, which is also an issue in the non-forensic mental health care (19, 37). Assistance in the form of family interventions have proved to reduce families’ burdens and be helpful in gaining control over their situation in the non-forensic mental health care, Nonetheless, the implementation of family interventions within forensic psychiatric care remains both infrequent and seldom focused in research projects (38).

The telephone is highlighted as a very important tool in the pursuit of maintaining relationships despite long distances and security restrictions. This study underscores a significant distinction between having access to a personal mobile phone versus solely having access to a landline. The possession of a mobile phone expands the avenues for contact, enabling communication to extend throughout the day. Despite the recognized importance of both telephones and mobile phones in preserving family relationships, there is a dearth of comparable studies emphasizing these aspects in such an important manner. A regulatory change regarding the use of mobile phones was introduced in Sweden in 2014 based on a government proposition (39), which restricted the possession of personal mobile phones to only those wards with the lowest security classifications. This decision was prompted by the awareness of the risks associated with mobile phones, including the potential for patients to engage in criminal activities or misuse the device for inappropriate purposes. The risk perspective on mobile phones in forensic psychiatric care currently outweighs their potential benefits for maintaining family relationships, such as the convenience of easy-access video calls and social media communication.

In accordance with a study by Rowaert (18), families often describe themselves as powerless, facing significant difficulties in influencing the situation or providing support to one another. However, the mutual support that families can offer each other proves to be of significant importance, sometimes even outweighing the assistance provided by healthcare professionals. The importance of maintaining good contact with family members for the patient’s well-being has also been reported in other studies (10, 11). However, this study highlights a tangible reciprocity, emphasizing that families should be viewed as units requiring help and support to recover. The families in this study described themselves at times as being broken, incomplete, and lacking the resources to support each other effectively. They also spoke of a dependence on the support and guidance of healthcare professionals to understand and cope with situations of uncertainty. This underscores the need for a holistic approach that recognizes the family as a crucial component in the patient’s recovery process.

It has been found in other studies (34, 40, 41) that family members often spoke of ambivalence in their feelings toward the patient, particularly due to the patient’s crime and illness thus making it challenging to relate to them as they had done previously. This is in stark contrast to the results of the present study, where none of the family members spoke of ambivalence toward the patient or hesitated to provide assistance. This difference may stem from the fact that the patients themselves determined who constituted their family during the interviews. Those who participated may have refrained from talking about ambivalence in front of the patient, or the composition of the family may have changed, with the patient selecting members who still provided support. Whatever the cause for this the present study does not support the notion that the individuals who the patient identifies as belonging to their family harbor ambivalence in their feelings toward the patient or their situation. Instead, all family members expressed a desire for the patient’s well-being and recovery.

## 6 Strengths and limitations

One of the strengths of this study is the diverse composition of the families included, encompassing variations in the duration of care, being cared for according to different security classifications and variations in gender, age and home area. This diversity generated a broad spectrum of experiences and perspectives, which enriched the data and facilitated a more comprehensive understanding of the phenomenon. Additionally, the use of thematic analysis according to Lindberg et al. (23) was particularly appropriate for the interview material, as it allowed for the identification and exploration of patterns of meanings and recurring themes within the data. The Reflective Lifeworld Research (RLR) approach according to Dahlberg et al. (29), which is the ontological and epistemological foundation for the used thematic analysis (23), further enabled a greater understanding by focusing on the meanings and experiences of the participants, thus capturing their lived experiences. This method provides a phenomenological and theory-grounded approach, which further strengthens the validity of the study in contrast to a thematic analysis. Using a phenomenon-oriented approach is demanding for the researchers, particularly in terms of controlling their own preconceptions. The process has been characterized by a reflective and “bridled” attitude with a deliberate effort to slow down understanding, ensuring the phenomenon was not understood and defined too quickly. However, the potential issue of pre-understanding is not necessarily seen as a limitation, as pre-understanding can also provide a starting point for exploration, while pre-understanding is temporarily “bridled”, it can later inform interpretation and enrich the understanding of the phenomenon being studied.

A richer and more varied data could have been achieved by conducting more interviews and including a wider range of family configurations, e.g. including individuals from diverse backgrounds, genders, and a wider range of family constellations, such as families with a greater number of actively involved members, which could also have enhanced the methodological rigor of the study. This approach could ensure a more comprehensive understanding of the phenomenon by capturing varied lived experiences and family dynamics, thereby increasing the study’s transferability and relevance. This would have enhanced the depth and breadth of the findings, allowing for a more nuanced exploration of the diverse experiences and perspectives. Moreover, a larger sample size might have increased the generalizability of the results, providing a more robust foundation for drawing conclusions and making further recommendations. The phenomenon-oriented approach to the concept of “family” allowed the patient to define who was included in their family, ensuring that the study remained true to the phenomenon. However, this definition of family is crucial to consider when reflecting on the study’s relevance within the research field.

## 7 Conclusion

A family with a family member who is being cared for in forensic psychiatric care have support needs that are not fulfilled, even though these have been highlighted from several different perspectives. It is crucial to emphasize the importance of adopting a family-oriented practice in forensic psychiatric care, even in situations where the family is dysfunctional. Healthcare professionals might be able to provide substantial support to the family by using simple yet effective methods, such as active listening and empathetic communication. By fostering an open dialogue and understanding, caregivers can help bridge gaps and facilitate better outcomes. A thoughtful and compassionate approach can create a supportive environment that benefits everyone involved even when family relationships are strained or complex. The quality of care provided can be significantly enhanced by ensuring that healthcare professionals are equipped with the skills to navigate these delicate situations. A family-oriented practice should thus become a fundamental component of caregiving strategies in forensic psychiatric care. Our suggestion is that education focusing on a family-oriented practice emphasizing the importance of an open and empathetic dialogue, should be provided for those working in forensic psychiatric care, particularly nurses and members of staff in close contact with the patients. Those educational interventions should be tailored for the forensic psychiatric care context with an understanding for the complex nature of the care provided and the family dynamics.

## Data Availability

The datasets presented in this article are not readily available due to ethical considerations. Even though the interviews are anonymized, ethical guidelines restrict their access exclusively to researchers involved in the project. Requests to access the datasets should be directed to gustav.bjornberg@lnu.se.

## Footnotes

1 There is a role known as a support person in forensic psychiatry and coercive legislation in Sweden. The support person serves as a compassionate point of contact for the patient during their hospitalization and provides civic oversight in an otherwise closed psychiatric healthcare system. All patients have the right to have continued contact with their support persons (5, 6).

2 Telephones are provided that the patients can borrow in security class 1 and 2 in forensic psychiatric inpatient care. The patient is usually obligated to state the number they want to call and then the staff dials the number before handing over the phone to the patient. Patients are allowed to have their own mobile phone devices in security class 3 and forensic psychiatric outpatient care.

3 Security levels in forensic psychiatry in Sweden vary, and there are different security classifications: security classes 1, 2, and 3. Security class 1 is maximum security, security class 2 is high security, and security class 3 is acceptable security. The chief medical director determines the security classification, and their assessment is based on the perceived dangerousness of the patient (SKR, 2022; SFS 1991:1129).

4 In Swedish forensic psychiatry, patients can be referred to a regional clinic, which is a specialized forensic psychiatric setting that can provide a more specialized forensic psychiatric care that is not available in the patient’s home county. There are five regional clinics, and those can be located far from the places where the patients have their homes (SKR, 2022).
